# Experimental studies on effects of temperature on oil and water relative permeability in heavy-oil reservoirs

**DOI:** 10.1038/s41598-018-31044-x

**Published:** 2018-08-21

**Authors:** Yadong Qin, Yongbin Wu, Pengcheng Liu, Fajun Zhao, Zhe Yuan

**Affiliations:** 1 School of Energy Resources, China University of Geosciences(Beijing), 29 Xueyuan Road, Beijing, 100083 China; 2 Key Laboratory of Marine Reservoir Evolution and Hydrocarbon Enrichment Mechanism, Ministry of Education, 29 Xueyuan Road, Beijing, 100083 China; 3 Research Institute of Petroleum Exploration and Development, PetroChina, 31 Xueyuan Road, Beijing, 100083 China; 4 Key Laboratory of Oil Recovery Enhance of Ministry of Education, Northeast Petroleum University, 199 Fazhan Road, Daqing, 163318 Heilongjiang China

## Abstract

A heavy-oil sample derived from a block of Venezuelan oil was used to investigate effects of temperature on relative permeability to oil and water. Measurements of relative permeability were based on one-dimensional core-flow simulated systems using an unsteady-state technique at different temperatures, and then impact rules of temperature dependency were discussed. Both water and heavy oil in cores were reconfigured under the consideration of actual reservoir conditions. Study results suggest that relative permeability is high to oil phase and is very low to water phase, and fluid flow capability is extremely imbalanced between oil and water. As temperature increases, irreducible water saturation linearly increases, residual oil saturation performs a nonlinear decrease, and water saturation exhibits a nonlinear increase at equal-permeability points. The water-wettability of rocks is heightened and overall relative permeability curves shift to the right with increasing temperature; furthermore, two-phase flow area becomes wider and both oil and water relative permeability increases apparently, but the increase ratio of water is less than that of oil.

## Introduction

Relative permeability to heavy oil and water is one of the most important parameters of production and prediction in oilfields. Inputting variables for numerical reservoir simulation models and characterizing underground flow behavior of immiscible fluids are two very critical applications of relative permeabilities. Heavy-oil reservoirs are mainly thermal recovery reservoirs; thus, heavy oil is very sensitive to temperature; hence, researching change regulations of oil-water relative permeability of heavy oil for differing temperatures is becoming extremely significant^1–4^. At present, numerous related experiments on heavy oil and water relative permeability are based on displacements of dead or refined oil by distilled water. The authors of these studies did not consider dissolved gas in oil and salinity in water under the conditions of practical reservoirs, which may create certain deviations upon oilfield application. Moreover, controversy still surrounds the issue of whether relative permeability is dependent or not on temperature; no consensus has yet been reached.

In the 1960s, measurements of relative permeability with increasing temperature began to appear in thermal oil recovery studies. Every researcher has drawn their own conclusions in more than 50 years of investigation, and two conflicting statements have emerged. Some researchers report that relative permeabilities are dependent on temperature, and provided impact rules^5–20^, but others concluded that temperature has little effect on relative permeability or that no direct influences exist^21–29^. A majority of the studies asserted that both statements provided a basically agreed-upon review of a decrease in residual oil saturation with increasing temperature^5–10,22,23^. Regarding the performance of irreducible water saturation, a typical increase with temperature is found; nevertheless, according to Sun’s observations, irreducible water saturation increases initially and then decreases with increasing temperature^10^. Sun attributed this phenomenon to the volume expansion of water caused by elevated temperature, and he concluded that the expansion affects dominantly occur in irreducible water at temperatures greater than 250 °C.

Many different observations of temperature-effect rules related to relative permeabilities of oil and water phases exist. Both Edmondson and Davidson reported that the water-oil relative permeability ratio exhibits sensitivity to temperature at low and high water saturation, but insensitivity at moderate saturation^5,6^. Lo and Mungan indicated that relative permeability to oil increases with increasing temperature, but the increase rate of water relative permeability presents more in both water-wet and oil-wet systems^8^. Bennion found that relative permeability to oil and water increases with increasing temperature when less than 100 °C^11,12^. Akhlaghinia observed that permeability of oil phase in an experimental reservoir increases to a maximum and then decreases with increasing temperature^16,17^. Experimental results obtained by some researchers revealed no reduction, and at most few changes, in both oil and water relative permeability with increasing temperature, so they concluded that oil-water relative permeabilities are independent oftemperature^21,24–28^.

Many researchers have demonstrated that there is no direct relationship between temperature and relative permeabilities. A segment of them found the existence of change rules in endpoint saturation and relative permeabilities in their experiments, but they attributed those rules to the viscosity ratios of oil-water changing with temperature^21,24–26^. Indirect influences of temperature on relative permeabilities include changes of viscosity, thermal expansion, and transitions of rock properties, such as wettability and construction with temperature. Long *et al*. observed a phenomenon during their tests in which residual oil saturation decreases by only 4%, from 60 °C to 200 °C, which is a very small decrease of residual oil saturation with such a great increase in temperature. In addition, they agreed that the phenomenon demonstrates that the reasons are thermal expansion and transition of wettability^26^. In addition, Ashrafi *et al*. concluded that the temperature dependence of relative permeabilities is related more to experimental artifacts, viscous fingering, and viscosity changes than to fundamental flow properties. Measurement errors, artifacts, and calculations are inevitable in experiments and tests, and, as a result, some studies have asserted that these inevitable factors lead to temperature effects on relative permeabilities^22,23^.

It can be observed that there is no consensus regarding effects of temperature on oil-water relative permeability. Some studies have shown that relative permeability depends on temperature, but others disagreed with this by attributing changes to experimental errors, viscous instabilities, or variation of rock properties. Furthermore, in most of the aforementioned studies tests were conducted that were based on displacements of dead or refined oil by distilled water, which could lead to the existence of certain deviations in oilfield application. Furthermore, impact rules revealed by these studies regarding equal-permeability points are inadequate, and only briefly describe that equal-permeability points shift to the right or exhibit nearly no variation with temperature without detailed analysis. A Venezuela heavy oil sample was used in this study, and live oil and salty water were reconfigured under the consideration of actual reservoir conditions. The relative permeability was measured using an unsteady-state technique at different temperatures (45 °C–200 °C). An attempt was made in this research to obtain results meaningful to the development of heavy-oil reservoirs and to achieve a correct understanding of the effects of temperature on oil-water relative permeability.

## Experiments

### Experimental apparatus

**Figure 1:**
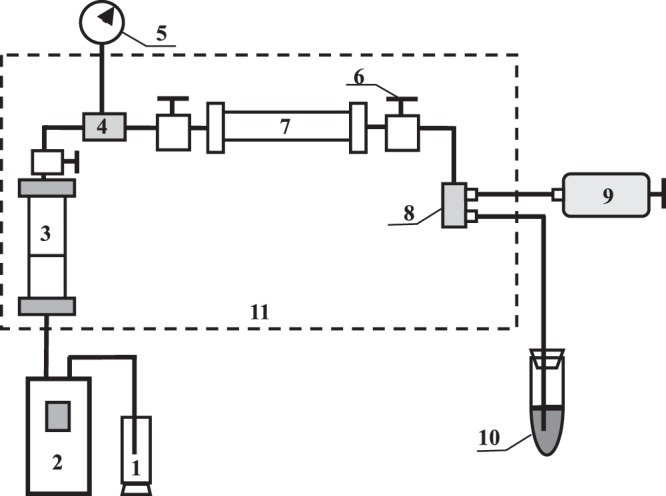
Schematic diagram for experimental evaluation of heavy oil relative permeability. 1-pump working fluid; 2-ISCO pump; 3-intermediater container; 4-pressure sensor; 5-pressure gauge; 6-valve; 7-sand pack; 8-backpressure regulator; 9-hand pump; 10-oil-water collector; 11-thermostat oven.

The experimental apparatus mainly consists of four parts: an injection system, physical model system, pressure measurement and control system, and outlet liquid measurement system. The flow diagram of the experimental apparatus is shown in Fig. 1. The injection system includes an International Standard Classification of Occupations (ISCO) pump and an intermediate container. The ISCO pump is used to drive the fluid into the physical model system and to pressurize the system at a constant pressure or constant flow rate. The maximum flow rate is 30.0 ml/min and the maximum pressure is 70.0 MPa. The pump working fluid is distilled water. A live oil sample is transferred by the intermediate container to the physical model system. A backpressure regulator adjusted by a hand pump, whose maximum operating pressure is 40.0 MPa, is used to control pressure at the outlets of the sand packs. An electronically controlled thermostat oven with a maximum temperature of 250 °C is used to ensure a constant temperature in the range of experimental temperatures from 45 °C to 200.0 °C. An oil-water collector is used to record the amount of water and oil production. The experiments do not require any gas volume gauges because the amount of dissolved gas can be negligible, and the density of live oil was obtained prior to the experiments.

### Material

There were four sand packs, all comprised of 60-/70-mesh grain-size quartz sands derived from a block of Venezuela oil. The sands were packed into 2.5-cm-diameter and 30.0-cm-long stainless-steel tubes. All the physical parameters are listed in Table 1.

**Table 1:** Physical parameters of four sand packs used in experiments.

Number	Length (cm)	Diameter (cm)	Permeability to air (mD)	Porosity (%)
1	30.0	2.5	5560.00	47.69
2	30.0	2.5	4139.54	43.21
3	30.0	2.5	4972.33	43.86
4	30.0	2.5	5096.09	45.45

**Figure 2:**
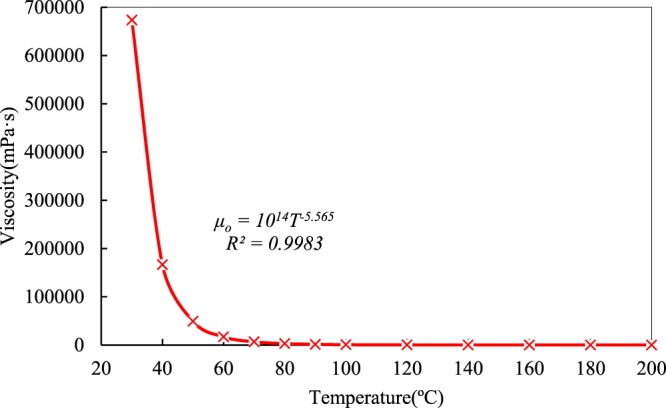
Viscosity-temperature curve of dead heavy oil.

**Figure 3:**
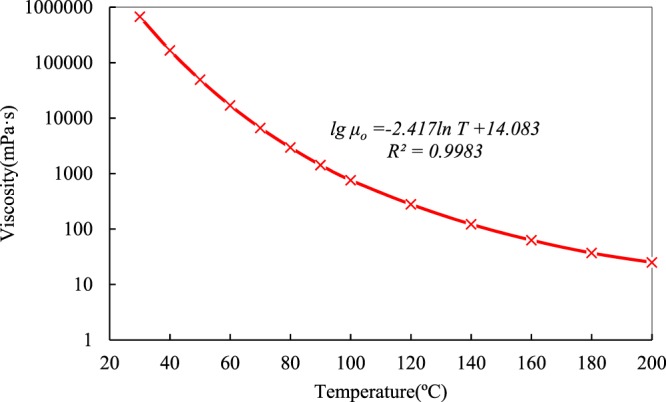
Semi-logarithmic viscosity-temperature curve of dead heavy oil.

The dead heavy oil sample was derived from the block of Venezuela oil, and before the experiments, live heavy oil was configured under practical reservoir conditions, which is a dissolved-gas-to-oil ratio of 8.9 m^3^/m^3^ at a temperature of 45 °C and pressure of 4.2 MPa. The dissolved gas is comprised of 99.0% methane, 1.0% other gases. The relationship curve between the viscosity and temperature of dead oil is shown in Fig. 2, and the semi-logarithmic viscosity-temperature curve is shown in Fig. 3. The viscosity-temperature curve of the dead heavy oil illustrates an extremely high viscosity at lower temperature and the entire curve exhibits a power function with a large coefficient of 14 orders of magnitude. The dead heavy oil is very sensitive to temperature, and its viscosity value is 63077.19 mPa·s at 45 °C and then decreases to 1424.9 mPa·s at 90 °C.

It can be observed that the curve in Fig. 3 is not linear and its trend become slows with increasing temperature. An order of magnitude decrease in viscosity value from 673650.1 mPa·s at 30 °C to 63077.19 mPa·s at 45 °C is observed, while the temperature only increases by 15 °C. However, with increasing temperature from 90 °C to 140 °C, an increase of 50 °C, an order of magnitude decrease in viscosity value, from 1424.9 to 122 mPa·s, is observed. As the temperature becomes greater than 90 °C, the dead heavy oil gradually become insensitive to temperature despite its high viscosity at low temperature.

Owing to the fact that gas was dissolved into the dead oil, the viscosity changed. The live-oil viscosity was obtained by an empirical formula method in the absence of experimental measurements. The relationship between live and dead oil was proposed by Beggs and Robinson in 1975^30^. The empirical formula is

1 $${\mu }_{o}=A{\mu }_{od}^{B},$$

where

2 $$A={\rm{10.715}}\times {({R}_{so}+100)}^{-0.515},$$

3 $$B={\rm{5.44}}\times {({R}_{so}+{\rm{150}})}^{-0.338},$$

where *R*_*so*_ is the ratio of dissolved gas to oil, in scf/STB; *μ*_*o*_ is the viscosity of live oil, in mPa·s; and *μ*_*od*_ is the viscosity of dead oil, in mPa·s.

However, the empirical formula proposed by Beggs and Robinson was originally used for calculating the viscosity of heavy oil with dissolved gas under reservoir conditions, and it did not take into account the fact that the density of live heavy oil varies with temperature. A new empirical formula was adapted by Yang *et al*., as^31^

4 $${\mu }_{o}=A{\mu }_{od}^{B},$$

where

5 $$A=4.4044\times {({\rho }_{osc}{R}_{so}+17.7935)}^{-0.515},$$

6 $$B=3.0352\times {({\rho }_{osc}{R}_{so}+26.6904)}^{-0.338},$$

where *ρ*_*osc*_ is the density of dead oil on the surface, in g/cm^3^; *R*_*so*_ is the ratio of dissolved gas to oil, in m^3^/t; *μ*_*o*_ is the viscosity of live oil, in mPa·s; and *μ*_*od*_ is the viscosity of dead oil, in mPa·s.

The viscosity of dead oil is input into formula (4) to calculate the viscosity of live oil, and the four groups of new viscosity values obtained are listed in Table 2.

**Table 2:** Viscosities of live-heavy oil.

Temperature (°C)	45	100	150	200
Viscosity (mPa·s)	19799.95	358.02	44.96	16.09

The experimental water used was simulated connate water from actual reservoirs with total dissolved solids of 11582.6 mg/L, as shown in Table 3.

**Table 3:** Total dissolved solids in experimental water sample.

Cations	Na^+^	Ca^2+^	Mg^2+^	Ba^2+^	Fe^2+^	Total (mg/L)	
Ion concentration (mg/L)	3967.2	76	29.2	2	0.2	4074.6	
Anions	Cl^−^	SO_4_^2−^	CO_3_^2−^	HCO_3_^−^	OH^−^	Total (mg/L)	Total dissolved solids (mg/L)
Ion concentration (mg/L)	4710	16	0	2782	0	7508	11582.6

### Experimental procedures

The unsteady-state technique was used in this study to measure the oil-water relative permeability curves of heavy oil owing to its simplicity. Calculations of relative permeability were performed in software into which the Johnson,-Bossler-Naumann (JBN) technique was written.

The experimental procedures were as follows:

1. Before starting the experiments, the volumes of live oil and salt water were sufficiently configured in advance. Live oil was prepared in a sample-configuration container by mixing gas and dead oil at a solution gas-oil ratio (GOR) of 8.9 m^3^/m^3^ at a constant temperature of 45 °C and a designated pressure of 4.2 MPa, and then transferred to the intermediate container. The intermediate container filled with live oil was placed into a thermostat oven, which generated a constant temperature of 45 °C. The salt water was sealed up and placed in the shade.
2. Four identical stainless-steel tubes (2.5 cm in diameter and 30.0 cm long) were prepared and cleaned. 60-/70-mesh grain-size quartz sand was packed into the tubes and tamped down. The sand packs were then marked in a sequence.
3. After finishing the sand packs, the absolute permeability was measured and the sand packs were then vacuumized for 2.0 h, at which point the absolute permeability requirements of the experiments were met. Later, the cores were saturated with the simulated connate water. The porous volumes of the four cores were directly read out by a hand pump with a scale, which avoided errors in porosity calculations. During this process, the porous volumes of the four cores were read out when the pressure difference became steady between both ends.
4. Next, the sand packs were saturated by heavy live oil at differing temperatures (45, 100, 150, and 200 °C) to form irreducible water-saturation conditions. The saturation was halted when the volume of produced oil was 2–3 times that of the water produced at the sand-pack outlets, and the pressure drop became stable between the inlets and outlets of the cores. After saturation, the cores were placed in the thermostat oven at constant temperatures of different experimental groups for 24 h.
5. Oil-displacement experiments were conducted with a water-injection rate of 0.1 ml/min at 45 °C, and the injection rate was switched to 2.0 ml/min when experimental temperatures were higher than 100 °C. The monitored and recorded data exhibited pressure drops across the cores and an accumulation of produced oil and water over time. The experiments would end when the water cut was over 99.5%, the injected volume of simulated connate water reached 30 PV (porous volume), and the pressure drop became steady. The backpressure regulator was kept a pressure of 4.2 MPa by a hand pump in the processes of saturation and displacement to simulate the pressure environment of actual reservoirs.

## Results and Discussion

### Effects of temperature on irreducible water saturation and residual oil saturation

**Figure 4:**
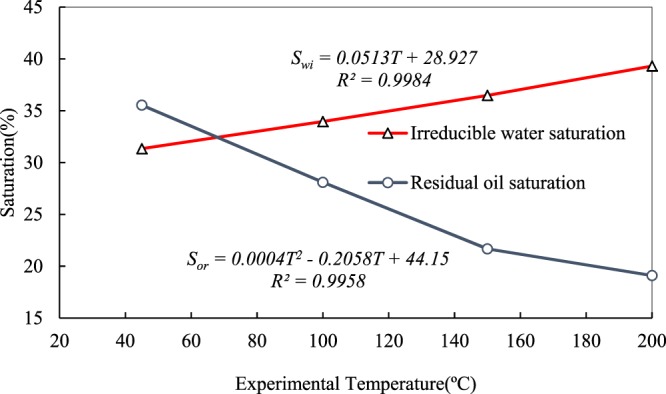
Irreducible water and residual oil saturation at different experimental temperatures.

Curves plotting irreducible water and residual oil saturation against experimental temperature are shown in Fig. 4. The irreducible water saturation linearly increased from 31.34% at 45 °C to 39.31% at 200 °C with an average increase of 2.66% per 50 °C.

Water phase with small viscosity is piston-displaced by the heavy oil, the viscosity of which is high at low temperature, which results in a low irreducible water saturation. With increasing temperature, expansion of rock particles makes micro-pores smaller and blocks the throats between small and large pores. Therefore, water droplets saturated into small pores are not easy to displace. Moreover, the viscosity ratio of oil to water decreases because the viscosity of heavy oil greatly decreases with decreasing temperature. Furthermore, the mobility ratio of oil to water increases, which leads to an increase of the flow capability of oil phase as a displacing phase. The surfaces of pores initially adsorb a great quantity of polar molecules, such as resin and asphalt, but they are desorbed gradually with increasing temperature. A significant number of water molecules are adsorbed as the thickness of the resin layer decreases. This case represents formation and gradual thickening of the water film. As a result, the irreducible water saturation exhibits an increase in cores.

Although irreducible water saturation increases linearly in the experimental temperature range, if temperature increases to more than 200 °C or even higher, the irreducible water saturation may increase to a peak value and then decrease as Sun reported. Sun also concluded that the volume expansion of water primarily leads to this reduction^10^. In fact, the mechanisms that lead to changes in irreducible water saturation at elevated temperature not only induce the volume expansion of water, but also include the desorption of water molecules and volume expansion of rocks. The desorption function reduces the number of water molecules on the surfaces of pores and leads directly to the decrease in irreducible water saturation. Moreover, the pressure generated by volume expansion of both water and rocks induces the production of more water.

The decrease of residual oil saturation is nonlinear with a functional quadratic relationship, while the maximum residual oil saturation is 35.53% and the minimum is 19.1%. The viscous fingering phenomenon is evident at low temperature during the displacement process, which is the major reason that water cut increases rapidly after breakthrough. Oil droplets that occupied small pores cannot be displaced, which results in higher residual oil saturation. The viscosity of heavy oil decreases significantly and the mobility ratio of water to oil decreases with increasing temperature. An increase in the sweep area of water diminishes the function of viscous fingering to some extent, and thus more oil is produced at the outlets.

The residual oil saturation decreases gradually due to the exponential decrease in the viscosity of heavy oil with increasing temperature (Fig. 2), and a decrease gradually reduces the viscosity ratio of oil to water. Resin and asphalt films thin as temperature rising, which makes further efforts to improve oil production. As the temperature increases, the flow capability of oil cannot increase endlessly in the experimental temperature range and the increase will finally decrease to zero. Therefore, the trend of residual oil saturation is a slow nonlinear decrease in the range of experimental temperatures.

### Effects of temperature on water saturation of equal-permeability points

**Figure 5:**
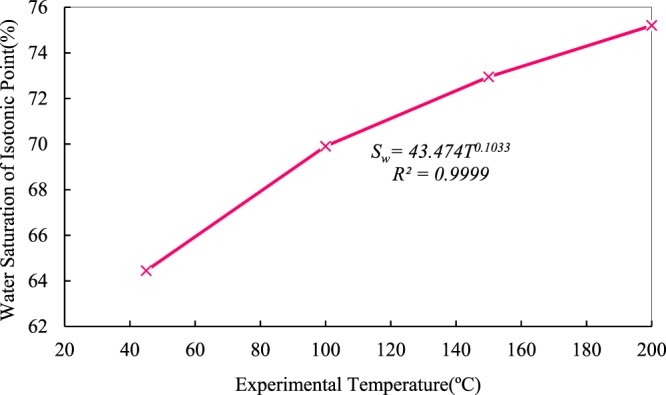
Relationship between saturation of equal-permeability points and experimental temperatures.

At equal-permeability points, relative permeability of water phase is equal to that of oil. The curve in Fig. 5 shows the relationship between water saturation of the equal-permeability points and residual-oil endpoints. The red curve, which shows that with increasing temperature, the water saturation of equal-permeability points increases nonlinearly and that the entire curve is a function of power. At 45 °C, the water saturation of the equal-permeability point is 64.45%, and it reaches 75.20% at 200 °C. The water-wettability of rocks is strengthened at elevated temperature, and it is already initially strong at low temperature. The transition of wettability reveals that high temperature gives rise to adsorption function of water molecules and changes of rock properties.

The water saturation of equal-permeability points increases gradually with increasing temperature, and the difference values of water saturation exhibit an increase between residual oil endpoints and equal-permeability points. The value is 0.02% at 45 °C, and it can be observed in Fig. 5 that the equal-permeability point and the residual oil endpoint almost coincide, and the difference reaches the maximum of 5.70% at 200 °C. The blue curve is a trend of water saturation at residual-oil endpoints, which avoid discussing in this section because details about residual oil saturation have been introduced in the previous section.

As the viscosity of heavy oil decreases with increasing temperature, the flow capability of oil phase increases, and hence it acts as a diminishing obstruction to water. Then, it can be inferred that the trends exhibit a decrease in the viscosity ratio of oil to water and an increase in water relative permeability. The curves of both phases shift to the right. Therefore, the equal-permeability points move to the right and an increase results from the water saturation of equal-permeability points.

However, water molecules became increasingly more active with increasing temperature, so the thickness of water film cannot become thicker indefinitely on the surfaces of pores, and the thickening ratio cannot remain unchanged or even become larger with increasing temperature. Moreover, the increase in oil relative permeability is larger than that in water relative permeability, which eventually leads to a small increase in the water saturation of equal-permeability points. In addition, adsorption will consequently be destroyed by high temperature. Thus, the water saturation of equal-permeability points will increase to a maximum and then decrease; that is, the equal-permeability point would first move to the right and then shift to the left.

### Effects of temperature on water relative permeability at equal-permeability points and residual oil endpoints

**Figure 6:**
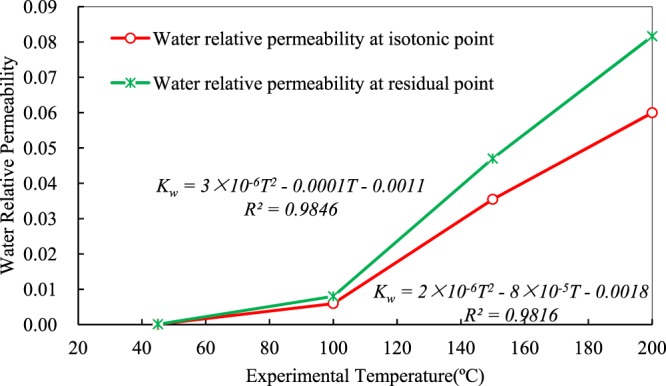
Relationships between water relative permeability and experimental temperatures at equal-permeability points and residual oil endpoints.

Relative permeability to water increases nonlinearly at both equal-permeability points and residual oil endpoints, as shown in Fig. 6. Both two curves are in quadratic function relationships. It can be found from the figure that the increase in the ratio of water relative permeability at temperatures from 45 °C to 100 °C is smaller than that at temperatures higher than 100 °C. It can be also observed in Fig. 6 that the relative permeability of water phase is extremely small at 45 °C, and the equal-permeability point almost coincides with the residual oil endpoint. Furthermore, at 200 °C, the water relative permeability is 0.0600 at the equal-permeability point and 0.0816 at the residual oil endpoint. This is because there is an inflection point near 90 °C in the viscosity-temperature curve of the heavy oil (in Figs 2 and 3), which is a watershed for the flowing capability of the heavy oil.

Before the turning point, the viscosity-temperature curve is very steep. Although the viscosity of heavy oil is extremely sensitive to temperature in this stage, oil phase exhibits poor flow capability with a thicker resin layer. Oil droplets generates the Jamin effect, which makes the flow of water more difficult, which can decrease the relative permeability with increasing temperature.

After the inflection point, the viscosity-temperature curve becomes gentle and the decrease of viscosity is slowly reduced, but the flow capability of oil phase is greatly enhanced. The hindrance function of oil phase decreases water flow owing to the thinness of the resin layer. The water-oil mobility ratio decreases and the flow capability of both water and oil increases with a large rise in relative permeability.

### Effects of temperature on relative permeability curves of heavy oil

**Figure 7:**
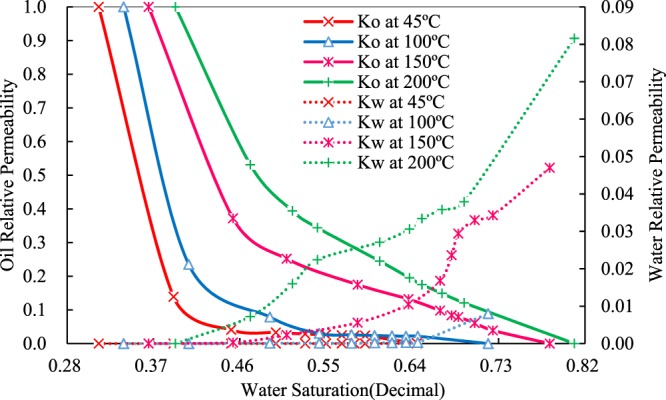
Relative permeability curves to oil and water at different experimental temperatures.

The different curves in Fig. 7 show the relative permeability to oil and water at experimental temperatures. The difference is dramatically large between oil and water relative permeability, indicating that relative permeability is extremely large to oil phase but very small to water phase.

There is almost no existence of two-phase flow region at 45 °C, and oil relative permeability decreases rapidly, while water relative permeability is nearly zero, which means that the heavy oil cannot be displaced at the reservoir temperature. The viscosity of the live heavy oil does not, however, reach the field mining criterion of 358.02 mPa·s at 100 °C, along with a small two-phase flow region. The decrease of oil relative permeability becomes gentle at 150 °C, at which the viscosity of the live heavy oil is 44.96 mPa·s. The curve of water relative permeability shifts upward significantly, along with exhibiting a larger two-phase flow region. It can be found that the decrease of oil relative permeability becomes slower at 200 °C than that at 150 °C, and a wider two-phase flow area, and a more significant uplift of the curve of water relative permeability, can be observed. It can be further observed that the relative permeability of both two phases improves remarkably with increasing temperature.

**Figure 8:**
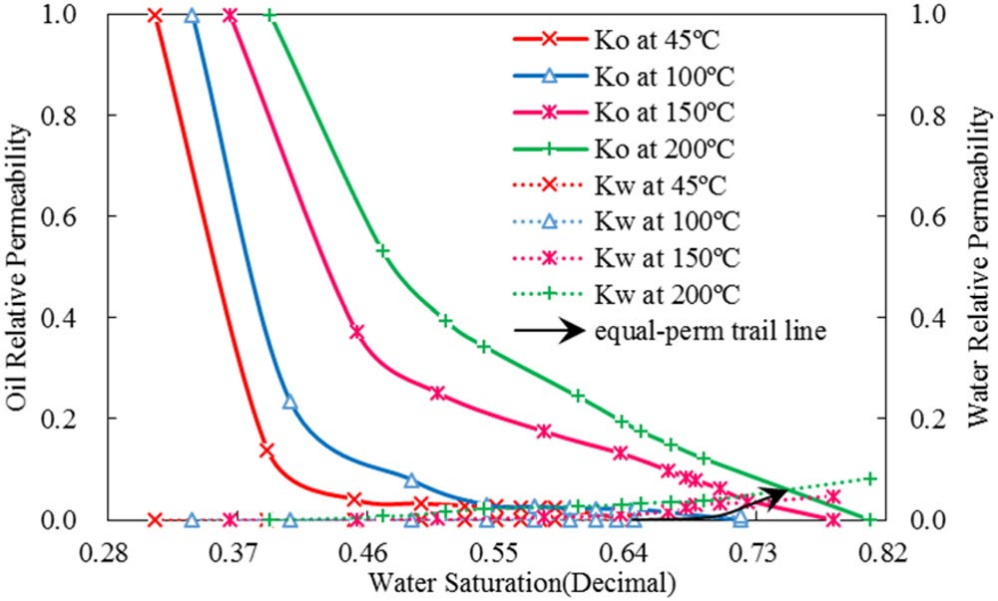
Relative permeability curves with the same scales to oil and water at different experimental temperature.

However, due to the extremely imbalance of relative permeability between water and oil, changes of the equal-permeability points cannot be observed easily. Furthermore, different scales are used in Fig. 7 presents an incorrect shift of equal-permeability points. And Fig. 8 shows the curves with the same scales are used. The black curve with an arrowhead exhibits changes of equal-permeability points with temperature. The equal-permeability point shifts to right evidently and raises upward visibly thanks to water relative permeability augments by various mechanisms.

Comprehensively, the rules of oil and water relative permeability with temperature are mainly rooted in three mechanisms: viscosity change of live heavy oil, adsorption and desorption function of fluid molecules, and thermal expansion of rock particles and fluid. Oil viscosity decreases with increasing temperature, flow capability of oil is enhanced, and the resin layer thickness decreases; hence, the hindrance function of oil phase to water phase decreases, which enhances the flow capability of water phase. However, meanwhile adsorption of water molecules becomes stronger with increasing temperature, which limits the mobility of water. As a result, the increase of the ratio of water relative permeability is smaller than that of oil relative permeability. Furthermore, as mentioned above, volume expansion of rock particles and fluid caused by elevated temperature generates expansion pressure to assist the production of fluid. This pressure contributes to the improvement of oil-water relative permeability.

## Conclusions

1. Flow capability of both oil and water improve and the relative permeability of both oil and water phase increase with increasing temperature. Relative permeability curves and the equal-permeability point shift to the right with temperature, and the equal-permeability point moves gradually further from the residual oil endpoint.
2. Irreducible water saturation increases linearly, and the decrease of residual oil saturation is nonlinear.
3. Although water relative permeability increases dramatically with temperature, it is still extremely different from oil. The relative permeability curves of two-phase flow area markedly increase with increasing temperature.
4. As the temperature increases, changes of characteristic points and oil-water relative permeability are essentially caused by mechanisms that include volume expansion of fluid and rocks, adsorption, and changes of oil viscosity. These factors are functions of temperature and, hence, relative permeability is also fundamentally a function of temperature.
